# Genetic Polymorphisms in Dopamine- and Serotonin-Related Genes and Treatment Responses to Risperidone and Perospirone

**DOI:** 10.4306/pi.2009.6.3.222

**Published:** 2009-08-03

**Authors:** Atsushi Tsutsumi, Tetsufumi Kanazawa, Hiroki Kikuyama, Gaku Okugawa, Hiroyuki Uenishi, Toshio Miyamoto, Naoki Matsumoto, Jun Koh, Kazuhiro Shinosaki, Toshifumi Kishimoto, Hiroshi Yoneda, Toshihiko Kinoshita

**Affiliations:** 1 Department of Neuropsychiatry, Osaka Medical College, Takatsuki, Osaka, Japan.; 2 Department of Neuropsychiatry, Kansai Medical University, Osaka, Japan.; 3 Department of Neuropsychiatry, Wakayama Medical University, Wakayama, Japan.; 4 Department of Psychiatry, Nara Medical University, Nara, Japan.; 5 Course of Letters, Kansai University, Osaka, Japan.

**Keywords:** Risperidone, Perospirone, Polymorphism, Clinical efficacy, Pharmacogenetic study

## Abstract

We investigated the possible association between genetic polymorphisms in the dopamine receptor and serotonin transporter genes and the responses of schizophrenic patients treated with either risperidone or perospirone. The subjects comprised 27 patients with schizophrenia who were clinically evaluated both before and after treatment. The genotyping of the polymorphisms of the dopamine D2 receptor gene (DRD2) (rs1801028 and rs6277), the dopamine D4 receptor gene (DRD4) (120-bp tandem repeats and rs1800955), and serotonin transporter gene (5HTT)(variable number of tandem repeats; VNTR) were performed using the real-time polymerase chain reaction and sequencing. In DRD2 and 5HTT-VNTR, there were no significant correlations between clinical response and polymorphism in the case of risperidone, and for perospirone treatment it was impossible to analyze the clinical evaluation due to the absence of genotype information. On the other hand, in DRD4 there were significant correlations in the two-factor interaction effect on the Positive and Negative Syndrome Scale (PANSS) between the two drugs [120-bp tandem repeat, p=0.003; rs1800955, p=0.043]. Although the small sample represents a serious limitation, these results suggest that variants in DRD4 are a predictor of whether treatment will be more effective with risperidone or with perospirone in individual patients.

## Introduction

Scores of antipsychotic drugs for treating schizophrenia are available, but it is difficult to anticipate which will the most effective in a particular patient because of the lack of biological indicators for their efficacies or the incidence rates of adverse effects. Several studies have investigated the effects of genetic polymorphisms on predicting treatment responses and the incidence of adverse effects. For the response to risperidone treatment, Xing et al.^1^ investigated 6 genetic polymorphisms in the dopamine D2 receptor gene (DRD2) in 125 Chinese schizophrenic patients. They reported that the A allele in the DRD2 A-241G single-nucleotide polymorphism (SNP) was associated with a higher risperidone efficacy. In addition, Wang et al.^2^ showed that an allele of the serotonin transporter gene (5HTT) reversing lipid transport (RLT) polymorphism may be a good predictor for a better outcome of risperidone treatment. However, Xuan et al.^3^ found that none of 8 SNPs in the dopamine D3 receptor gene (DRD3) were predictive in 130 schizophrenic patients taking only risperidone. The above studies are limited by only risperidone being considered, and hence the indicated genetic variants might be predictors for treatments with other drugs.

To assess which antipsychotic drug will be useful for a patient carrying a certain genetic variation, it is important to determine simultaneously which of several drugs are effective in numerous patients. Dolzan et al.^4^ investigated the effects of DRD1 A-48G, DRD2 Ins -141C Del, and DRD2 Ser 311 Cys variants on medical treatments of schizophrenia by risperidone, haloperidol, fluphenazine, or zuclopenthixole, and found no variations in the treatment response or the incidence of adverse effects. There are numerous positive and negative data on genetic variations in schizophrenia, and so the present study aimed to clarify these inconsistencies. The Schizophrenia Research Forum database (http://www.schizophreniaforum.org/)^5^ contains meta-analyzed data of all investigated variants published in peer-reviewed journals. Although the three variants investigated by Dolzan et al. are becoming accepted in genetic researches of schizophrenia, no individual variant is supported by the Schizophrenia Research Forum meta-analysis.

Our aim was to develop a reliable method for selecting antipsychotic drugs in individual patients. To this end, the involvement of genetic variations supported by the meta-analysis were studied for treatments by two serotonin/dopamine antipsychotic drugs: risperidone and perospirone. Perospirone is an antipsychotic drug that was developed in Japan;^6^ the profile of its receptor binding activity classifies it as a serotonin/dopamine antagonist (SDA).^7^ This agent is a 5-HT1A partial agonist and exhibits a higher efficacy in the treatment of anxiety^8^ and a lower incidence of hyperprolactinemia than risperidone.^9^ Although both drugs are classified as SDAs, it is suspected that they exhibit different affinities for dopaminergic and serotoninergic receptors that may underlie clinical variations in their efficacies or in the incidence rates of adverse effects. We therefore searched all of the genetic variants that were significantly supported by the Schizophrenia Database on Dopamine and Serotonin-Related Genes. The found variants were rs1801028 and rs6277 in DRD2, a 120-bp tandem repeats (TR) and rs1800955 in DRD4, and a 17-bp variable number of tandem repeats (VNTR) in 5HTT, and all of these were investigated in this study.

## Methods

The subjects were recruited from the cohort of patients who were treated in four Kansai medical organizations: Osaka Medical College, Kansai Medical College, Nara Medical University, and Wakayama Medical University. Other associated hospitals also cooperated in this study. All of the subjects were diagnosed as schizophrenic according to the International Classification of Diseases-10 (ICD-10) criteria by two trained psychiatrists, and informed consent was obtained from each subject. Each subject was randomly assigned to receive either risperidone or perospirone treatment. The drugs were allocated in a randomized double-blind manner. The Positive and Negative Syndrome Scale (PANSS) and the Drug-Induced Extrapyramidal Symptoms Scale (DIEPSS) were used for the clinical evaluation. The maximum treatment period was 3 months. It was possible to collect the clinical changes according to PANSS and DIEPSS from only 27 of the 39 patients who were originally enrolled in the study. This study was approved by the ethical committees at the four participating organizations and other associated hospitals. The entire study was conducted by the "Kansai four medical organizations", and the human rights of each participant were maintained throughout the treatment courses.

Each genomic variant was analyzed by the following methods: DNA sequencing for the 17-bp TR in the 5HTT gene, the restriction fragment length polymorphism (RFLP) polymerase chain reaction (PCR) method for the 120-bp TR, and the fluorescence resonance energy transfer (FRET) PCR method for the other three SNPs (rs1801028 and rs6277 in DRD2, and rs1800955 in DRD4). Experimental data from the analysis of each variant are reported in Table 1. Two-way analysis of variance was used to analyze the data (using SPSS, ver. 13).

## Results

The mean age of the 27 participants who were clinically assessed was 37.0 years, and 55.6% were male. The individual DNA samples were analyzed for Hardy-Weinberg equilibrium, and none of the individual markers of the five variants deviated significantly from the expected frequencies. Overall, the response as assessed by the PANSS did not differ significantly between risperidone and perospirone treatments (p=0.655). The results for each variant are presented in Table 2.

### D2 receptor gene (rs1801028, rs6277)

For the rs1801028 polymorphism, it was impossible to analyze the clinical evaluation with perospirone due to the absence of the A/G or A/A genotype within the perospirone-treated group. The change in the PANSS score in patients treated by risperidone did not differ significantly between A/G and G/G genotypes. A similar tendency was found for the analysis of rs6277, with all of the samples treated with perospirone being categorized as the C/C genotype. The PANSS score did not differ significantly between C/C and C/T genotypes among patients who received risperidone. For the investigated DRD2 polymorphisms, it was almost impossible to predict the clinical response to risperidone treatment.

### D4 receptor gene (120-bp tandem repeat, rs1800955)

**FIGURE 1:**
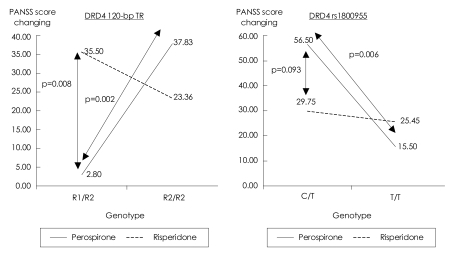
DRD4 120-bpTR: Within R1/R2 genotyped samples: risperidone vs. perospirone [F (1.22)=8.41, p=0.008]. Within perospirone-treated group: R1/R2 vs. R2/R2 [F (1,22)=11.85, p=0.002]. DRD4 rs1800955: Within perospirone-treated group: C/T vs T/T [F (1,23)=8.99, p=0.006]. DRD4: dopamine D4 gene, PANSS: Positive and Negative Syndrome Scale, TR: tandem repeat.

For the 120-bp TR, a two-factor interaction effect in the PANSS score was found to be significant between the two drugs (p=0.003, Figure 1). A significantly different two-factor interaction was also observed for rs1800955 (p=0.043, Figure 1) between the groups treated with perospirone and risperidone.

### 5HTT-variable number of tandem repeats

The clinical response to perospirone treatment tended to be better in 10/10 genotypes than 12/12 genotypes (10 and 12 referring to the number of base pairs repeated in the VNTR), but this difference was not statistically significant. The clinical response in the risperidone group did not vary with the genotype. An evaluation of genotype differences between the two agents also found no significant correlation.

## Discussion

This study has limitations in terms of the small sample and the absence of validation tests for the blood concentrations of the investigated drugs. Also, the statistical analysis was not completed for several variants due to the small number of observations, and hence the presented results should be interpreted with caution. Within the investigated five variants, only the 120-bp TR and rs1800955 were possible predictors of the clinical response to risperidone or perospirone. For the 120-bp TR and rs180095 in DRD4 there were clinical differences in the PANSS score between the two drugs. Patients with the 120-bp TR tended to respond well to risperidone especially in terms of the PANSS score for positive symptoms, whereas those with rs180095 tended to respond well to perospirone especially in terms of the PANSS anxiety score. Based on PANSS scores, it is strongly suggested that patients with the R1/R2 genotype (the combination of one repeat of 120-bp with two repeats of 120-bp) on the 120-bp TR should receive risperidone, and patients with the C/T genotype on rs1800955 should receive perospirone. Both the 120-bp TR and rs1800955 are reportedly related to the risk of schizophrenia onset, and a meta-analysis positively supported the results for the variants described herein.^5^ Located in the promoter region of the DRD4 gene, these variants appear in different haplotype blocks.^10^ In addition, the longer allele of the 120-bp TR and the T allele on rs1800955 decrease the DRD4 messenger RNA (mRNA) expression,^11^,^12^ so these variants are regarded to be genetically functional. A genetic study of schizophrenia found an association with the longer allele on the DRD4 gene, whereas the rs1800955 C allele-which may lead to higher expression of DRD4 mRNA-was associated with schizophrenia. Therefore, how the alteration of DRD4 expression relates to the etiology of schizophrenia remains controversial. In a postmortem study of schizophrenic brains, the expression of DRD4 mRNA was found to be three times higher in the prefrontal cortex.^13^ However, risperidone and haloperidol are known to increase the expression of DRD4,^14^ so further studies are needed to reveal those alterations in mRNA expression that are related to the pathogenesis of schizophrenia or the effect of antipsychotic agents.

Thus, we aimed to develop reliable criteria for choosing risperidone or perospirone for the treatment of schizophrenia according to the genetic characteristic of individual patients. As for the choice of different SDAs, although the small number of analyzed samples prevented us from determining whether the treatment efficacies of the two antipsychotics are influenced by the DRD4 genotype, our results suggest that an individual with the R1/R2 120-bp TR DRD4 genotype should be treated with risperidone, whereas one with the C/T genotype on rs1800955 in DRD4 should receive perospirone. Both of the genotypes (R1/R2 in the 120-bp TR, and C/T in rs1800955) indicative of perospirone treatment are known to increase the risk of schizophrenia. Future studies should investigate how perospirone regulates DRD4 in order to clarify the pathophysiological involvement of DRD4.

## Figures and Tables

**TABLE 1:**
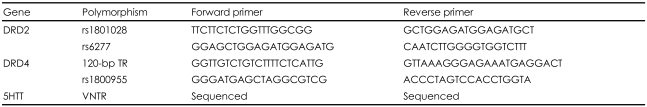
Genotyping details for single-nucleotide polymorphisms (SNPs) investigated in the DRD2, DRD4, and 5HTT genes

DRD2: dopamine D2 receptor gene, DRD4: dopamine D4 receptor gene, 5HTT: serotonin transporter gene, TR: tandem repeat, VNTR: variable number of tandem repeats

**TABLE 2:**
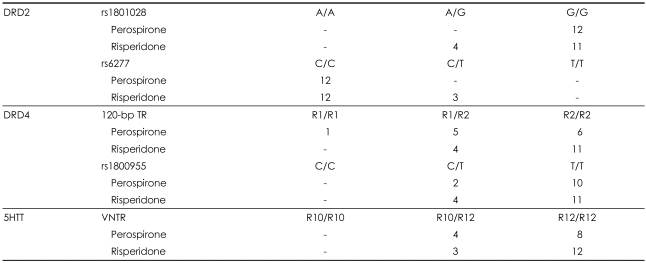
Allele frequencies for SNPs in the DRD2, DRD4, and 5HTT genes under perospirone and risperidone treatments

Numbers in the table represent the number of patients. DRD2: dopamine D2 receptor gene, DRD4: dopamine D4 receptor gene, 5HTT: serotonin transporter gene, TR: tandem repeat, VNTR: variable number of tandem repeats
